# How Personality Shapes Emotional Reactions to Explicit, Implicit, and Positive Media Images of Terror? An Experimental Investigation

**DOI:** 10.3390/ijerph22101581

**Published:** 2025-10-17

**Authors:** Tal Morse, Avi Besser, Virgil Zeigler-Hill

**Affiliations:** 1 Department of Photographic Communication, Jerusalem Multidisciplinary College, Jerusalem 91010, Israel; 2 The Centre for Death and Society, University of Bath, Bath BA2 7AY, UK; 3 Department of Communication Disorders, Jerusalem Multidisciplinary College, Jerusalem 91010, Israel; 4 Department of Psychology, Oakland University, Rochester, MI 48309, USA; zeiglerh@oakland.edu

**Keywords:** personality, terror attack, emotional reactions, exposure to media

## Abstract

This study investigates the public health consequences of media exposure to terrorism by examining individuals’ emotional responses to photographs from the October 7th terror attack, assessing how such imagery interacts with personality traits to influence emotional states. The research aims to explore how these reactions are moderated by personality traits—specifically the Big Five. A diverse sample comprising Israeli Jews (final sample *N* = 826) viewed media-sourced images categorized as explicit negative (*n* = 279; e.g., photos of bodies or deceased individuals), implicit negative (*n* = 269; images depicting destruction and devastation without explicit death symbols), and positive (*n* = 278; images of reconstruction and renewal). Participants’ affective states and specific emotions were assessed both before and after exposure to capture potential shifts. Results revealed a significant increase in negative emotions and a corresponding decrease in positive emotions following exposure to negative images. Personality traits moderated these emotional responses in nuanced ways. Neuroticism exacerbated negative emotional reactions, particularly among men exposed to implicit negative imagery, likely reflecting heightened sensitivity to ambiguous threats. Similarly, agreeableness was associated with heightened anger responses—specifically among men exposed to implicit negative imagery and women exposed to explicit negative images—although this effect was limited to anger and did not extend to other negative emotions. In contrast, openness was linked to decreased anger but only for men exposed to implicit negative imagery. Together, these findings underscore the complex interplay between media exposure, personality traits, and emotional responses to terror-related content. From a public health perspective, the results highlight the need for the following: (a) targeted mental health interventions that account for personality-based vulnerabilities, (b) responsible media reporting practices that minimize unnecessary harm, and (c) media literacy initiatives that empower individuals to manage exposure to distressing content. By linking personality, media imagery, and emotional outcomes, this study provides actionable insights for strengthening resilience, guiding ethical media practices, and promoting psychological well-being in communities affected by terrorism.

## 1. Introduction

Contemporary wars and terror attacks take place not only on the ground, but also in the media [1,2,3]. Accordingly, the toll of war and terrorism extends far beyond physical harm, exerting a profound impact on mental health across diverse populations. In the context of ongoing ethno-political conflict, many Israeli citizens are periodically exposed to traumatic events such as missile strikes, stabbing attacks, and suicide bombings. Over the past two decades, persistent security tensions with Hamas—particularly in the Gaza Proximity Zone—have resulted in ongoing threats and violence. By examining how individual psychology shapes responses to media exposure in the context of terrorism, this study highlights critical pathways through which traumatic events affect population emotional functioning, thereby advancing a transdisciplinary understanding of global public health challenges and informing strategies for resilience and prevention.

While previous research has examined the impact of media violence and personality traits on emotional responses [4], the present study uniquely investigates the nuanced interplay of personality traits with explicit and implicit negative imagery in the specific context of the October 7th terrorist attacks in Israel. This highly publicized and emotionally charged event took place in a saturated media environment, when terrorist organization bypass news organization and use technologies of self-documentation and decentralized platforms to disseminated unfiltered imagery from the scene of death. And indeed, the visual documentation that emerged from the October 7th attacks contained explicit depiction of slaughter and gruesome death imagery that almost never make it to the news [5,6]. Thus, this event provides a critical backdrop for understanding the psychological impact of exposure to violent media depiction within a population directly affected by terrorism. Our study extends the existing literature by exploring not only how emotional reactions vary according to the type of imagery (explicit negative, implicit negative, or positive), but also how these effects depend on individual differences in personality traits.

The Hamas assault on 7 October 2023, caused widespread devastation, triggering a humanitarian crisis and inflicting collective psychological trauma across affected communities [7,8,9,10]. This attack not only intensified existing mental health challenges but also underscored the urgent need for effective, targeted mental health interventions to support populations exposed to terrorism-related trauma. Understanding the psychological impact of exposure to media images of terrorism is crucial for developing effective public health strategies that promote resilience, protect mental health, and enhance community well-being. This study contributes to that goal by examining how personality traits moderate emotional responses to media portrayals of the October 7th attacks, providing insights that can guide targeted interventions and tailored support for affected populations. By identifying these psychological pathways, the findings inform health promotion initiatives designed to mitigate adverse mental health consequences and strengthen resilience in the face of terrorism and other traumatic events. These are all aligned with health promotion goals. Understanding how mediated exposure to terrorism affects individual psychology can inform health promotion efforts, encourage more responsible media practices and regulation, and enhance the work of mental health professionals.

This present study examines the emotional and psychological impact of exposure to media images depicting the October 7th attacks, with a particular focus on how individual differences in personality traits moderate these responses. We hypothesize that the visual depiction of terrorism—whether it emphasizes negative or positive imagery– can enhance or mitigate emotional responses, and that individuals with certain personality traits (e.g., high levels of neuroticism) will exhibit heightened emotional distress following exposure to negative media portrayals of the attacks.

### 1.1. Literature Review

The 7 October 2023 terrorist attacks in Israel provided a unique and critical context for the present study. This large-scale, highly publicized event generated widespread fear, uncertainty, and acute psychological distress across the population first and foremost due to brutal violence, but also by employing the presumed power of the visual to spread fear. Such events serve as powerful stressors that impact both individuals affected directly by terrorism and collective mental health of larger circles that were exposed to terrorism via the media. Existing research from Israel highlights the crucial role of socio-emotional factors—particularly personality characteristics—in shaping psychological responses to war and terror. These factors influence both immediate stress reactions and longer-term mental health outcomes [11,12] (for review see [13]) as well as PTSD symptoms, stress, and negative affect following exposure to traumatic cues [14]. Post-attack studies illustrate the extensive adverse psychological effects on various populations, including the general public and college students, manifesting as increased stress and reduced overall well-being [6,8,10].

The current study focuses specifically on the effects of exposure to negative photographs taken during the October 7th attacks and subsequently disseminated through the media. Research suggests that people are especially drawn to negative news content, as the potential costs of ignoring negative information often outweigh the perceived benefits of attending to positive news [15,16]. Negative news frequently highlights immoral behavior and the darker aspects of human nature, which can have adverse psychological and social consequences [15]. Moreover, negative content tends to spread more rapidly and reach a wider audience on social media platforms compared to positive or neutral content [17,18].

Despite the increasing prevalence of such media exposure, there remains a notable gap in the literature regarding the specific emotional consequences of viewing *visual* media images depicting acts of terrorism. Although some studies argue for a weak effect of visual materials on emotions and persuasion [19], others suggest that visual materials, especially photographs, are effective in swaying emotions and perceptions [20]. In particular, few studies have examined how individual differences—such as personality traits—may moderate emotional responses to this type of exposure. Although prior research has explored the effects of direct trauma and general psychological responses to terrorism, relatively little attention has been given to the nuanced impact of indirect exposure through media portrayals of death and violence, or to how these effects may vary across individuals.

The present study seeks to address this gap by experimentally examining emotional responses to both explicit and implicit media images depicting death related to sudden and unexpected terrorist attacks. We hypothesize that exposure to different depictive frames of terrorism—explicitly negative, implicitly negative, or positive—will elicit measurably distinct emotional responses from participants. We further hypothesize that exposure to such imagery will significantly affect individuals’ emotional states and that these effects will be moderated by personality traits. Understanding these moderating influences is essential for developing targeted mental health interventions and for informing media practices related to the dissemination of distressing content during and after terror incidents.

### 1.2. Exposure to Mediated Terrorist Related Images and Emotional Reactions

Research on the effects of exposure to media depictions of violence reveals complex interrelations between such exposure and emotional responses. A prominent example is the disparate reactions to imagery of the 9/11 attacks, which elicited celebratory responses among some audiences while generating fear and anxiety in others [21]. In a similar vein, Domke, Perlmutter, and Spratt (2002) argue that the common assumption that death images can inherently and independently shape public opinion is overly simplistic. They show that images are most powerful when they interact with a viewer’s pre-existing values, schemas, and political predispositions [22]. Joffe (2008) contends and argues that People interpret visuals differently based on identity, culture, and social positioning [20]. A meta-analysis of the effectiveness of images concluded that visual images—especially photographs—can evoke emotional responses, but negative images did not have significant effect on persuasion [19]. Powell et al. (2015) contend that despite studies about the effect of visual materials, less is known about how visuals evoke emotions that, in turn, influence opinions and behaviors toward issues [23]. Their study shows that images can have a strong emotional effect on audiences, but this can be mitigated by verbal framing.

According to appraisal theory, emotional reactions to situations or events are not automatic, but are instead based on how people interpret and evaluate them. Iyer and Oldmeadow (2006) showed that British participants who viewed newspaper images of a kidnapping victim in Iraq experienced significantly greater fear compared to those who did not see the images, but exposure to images did not increase sympathy with the victims or anger towards the perpetrators [24]. In a subsequent study, Iyer et al. (2014) demonstrated that exposure to images of terrorism increased levels of sympathy, anger, and fear, though the magnitude of these effects varied depending on whether the images depicted victims or perpetrators [25].

Empirical research further supports the connection between exposure to terrorism-related imagery and negative emotional reactions. For example, Huddy et al. (2003) found that Americans who consumed extensive media coverage of the September 11 attacks reported elevated levels of fear and depression [26]. Similarly, Lerner, Gonzalez, Small, and Fischboff (2003) demonstrated that exposure to terrorism-related photographs intensified emotional responses [27]. Similarly, studies demonstrate that exposure to violent media—particularly graphic imagery depicting death and trauma—can significantly heighten emotional arousal and psychological distress [28,29,30,31]. Viewers exposed to terrorist imagery often report increased feelings of fear and vulnerability, with some evidence suggesting that repeated exposure may lead to long-term adverse physical health outcomes [29]. Notably, even subtle or implicit cues associated with death can trigger subconscious emotional responses. A broad body of cognitive neuroscience research supports the concept of unconscious vigilance (for a review, see [32]), indicating that such cues can shape perceptions and attitudes toward terrorism without conscious awareness. These findings highlight the powerful psychological impact of violent imagery, which can activate threat and fear responses at both conscious and subconscious levels, ultimately influencing emotional regulation and cognitive appraisal. Such responses are of critical importance given their potential implications for both personal well-being and national security [28,33].

Deadly events do not always result in media coverage or in an abundance of images depicting death [34]. Yet, in today’s media environment, images depicting terrorist attacks—particularly those involving explicit or implied representations of death—have become increasingly common, especially on non-journalistic platforms, where ethics guidelines are not enforced [35,36,37]. These visuals often evoke strong emotional reactions, including fear, anxiety, anger, and sadness [38,39]. Although such images can raise public awareness and inform audiences, they also carry the potential to cause psychological harm, including heightened perceptions of threat, emotional distress, and symptoms similar to post-traumatic stress disorder (PTSD) [40]. The emotional toll of this content is evidenced by studies showing direct associations between media exposure and acute distress responses in the public [38].

Despite these consistent findings, questions remain about the psychological mechanisms that underlie these responses. Why do particular images elicit different emotional reactions—such as anger, fear, or sadness—and how do individual differences shape these outcomes? We argue that these responses are not only a function of the image content but are also moderated by dispositional traits such as personality. Appraisal Theory [41] provides a useful framework, suggesting that emotional responses are shaped by how individuals evaluate the relevance and meaning of an event. Similarly, Cultivation Theory [42] posits that repeated exposure to specific types of media content can alter an individual’s perception of social reality over time.

Understanding emotional responses to such images requires examining how viewers interpret and appraise what they see. These appraisals are shaped by both personality traits and situational contexts [40], as individuals assign meaning to visual content through interpretive processes that influence which emotions are activated [43]. As a result, two people may react to the same image with entirely different emotions depending on their psychological dispositions and the context in which the image is encountered. Accordingly, the present study aims to investigate how different depictions of terrorism affect the public and how personality traits influence emotional appraisals of media images related to terrorism, with particular attention to the role of individual differences in shaping these reactions.

In summary, the literature indicates that emotional responses to media depictions of violence are shaped not only by the content itself but also by individual interpretation [20] and pre-existing personality traits [12,14] (for review, see [13]). This complex interplay suggests that certain personality traits may make some individuals more vulnerable than others to the negative emotional effects of exposure to violent imagery. Furthermore, it underscores the importance of media outlets—including digital social media—exercising their influence responsibly to help minimize potential mental health consequences for viewers [44]. Building on these insights, the present study investigates how these factors jointly shape emotional reactions to media coverage of the October 7th attacks.

### 1.3. The Role of Personality Traits: Big Five Model

Individual differences in personality play a crucial role in shaping emotional and perceptual responses to media stimuli, including violent and trauma-related content [45]. The Big Five personality traits offer a well-established framework for understanding these variations [45,46]. Neuroticism, linked with heightened emotional reactivity and sensitivity to threat-related cues [47], tends to increase vulnerability to anxiety, fear, and emotional distress [48]. Openness, characterized by imagination and a willingness to explore new ideas, may influence how individuals interpret and respond to novel or potentially disturbing imagery [49]. Extraversion has been linked to greater emotional resilience, whereas Conscientiousness and Agreeableness may shape patterns of emotional reactivity and coping, potentially buffering against negative emotional outcomes.

These associations can be further explained by Appraisal Theory [50], which posits that emotional responses are shaped by individuals’ evaluations of events. For example, those high in neuroticism may appraise ambiguous or threatening images as more dangerous or distressing, resulting in intensified emotional responses.

Personality traits are generally understood as enduring patterns of thoughts, feelings, and behaviors that remain relatively stable across different stages of life and various situations [51]. Among the various models proposed to classify these traits, the five-factor model (commonly referred to as the Big Five) has gained broad empirical support [52,53]. Each trait reflects a distinct domain of psychological functioning. Neuroticism captures a tendency toward experiencing negative emotions such as fear, sadness, anger, guilt, embarrassment, and disgust, and is often associated with poor impulse control and hyperreactivity to stressors [52,54]. Extraversion refers to sociability, assertiveness, and a general tendency to seek stimulation and enjoy interpersonal interactions [55,56]. Openness encompasses imagination, creativity, esthetic sensitivity, and a willingness to explore novel ideas and experiences [55,57]. Agreeableness reflects compassion, cooperation, and trust in others, whereas conscientiousness involves self-discipline, organization, and goal-directed behavior [52,55].

A growing body of research supports the role of personality traits in predicting emotional responses to violent media. For example, individuals high in neuroticism report stronger distress and fear responses to violent or trauma-related content [48,58], whereas those high in extraversion or openness may display reduced emotional reactivity or respond with curiosity rather than distress. These personality-based differences are particularly relevant in the context of collective trauma, where understanding individual variation can inform psychological interventions and resilience-building strategies [51].

The Big Five traits function as core dispositional tendencies that exert a stable influence on behavior, emotion, and perception across contexts [59,60]. Consistent with this view, we hypothesize that heightened emotional responses to terrorism-related imagery are associated with higher levels of neuroticism and lower levels of extraversion, reflecting broader patterns of emotional sensitivity and resilience [58]. This idea is supported by findings from a study on Israeli civilian during the Al-Aqsa Intifada, which showed that both neuroticism and conscientiousness predicted stronger emotional reactions to political violence, whereas other traits did not exhibit significant effects [48]. Additionally, traits such as Openness and Agreeableness may shape how individuals interpret and engage with such imagery, influencing whether their responses lean toward fear, empathy, or reflective concern. Further, some research has suggested that terrorism-related media may heighten public awareness, possibly interacting with individual personality characteristics in complex ways [61].

### 1.4. Hypotheses

Drawing on these converging lines of research, the present study advances the following hypotheses: Given the well-established impact of negative imagery on emotional states among those who sympathize and identify with the victims, we expected that exposure of Israeli Jews to both explicit and implicit negative images of the October 7th attack would elicit greater emotional distress. However, we further hypothesized that these effects would be moderated by individual differences in personality traits:

**H1.**  *Neuroticism was expected to moderate emotional responses to the experimental conditions, such that higher levels of neuroticism will be associated with greater negative emotional reactions across all image types—particularly in response to explicit and implicit negative scenes. This hypothesis is based on evidence that individuals high in neuroticism are more sensitive to threat and tend to experience more intense negative affect in response to distressing stimuli, which we anticipate will be especially evident in the explicit and implicit negative conditions.*

**H2.**  *Conscientiousness was expected to moderate emotional responses to the experimental conditions, such that individuals higher in conscientiousness would display weaker negative emotional reactions to both explicit and implicit negative images. This attenuated response was anticipated based on prior research linking conscientiousness to enhanced self-regulation and goal-directed behavior, which may involve efforts to suppress emotional reactivity in favor of more pragmatic, solution-focused responses.*

**H3.**  *Extraversion was expected to moderate emotional responses to the experimental conditions, such that individuals higher in extraversion would exhibit weaker negative emotional reactions to both explicit and implicit negative images. This attenuated response was anticipated based on prior research suggesting that extraversion buffers against negative affect and promotes more positive emotional experiences, in part through the use of mood regulation strategies such as cognitive reframing and distraction.*

**H4.**  *Agreeableness was expected to moderate emotional responses to the experimental conditions, such that individuals higher in agreeableness would exhibit stronger negative emotional reactions to both explicit and implicit negative scenes. This hypothesis is grounded in evidence linking agreeableness with compassion, empathy, and concern for others, suggesting that higher agreeableness may be associated with greater empathic distress in response to distressing or emotionally evocative content.*

We did not formulate a specific hypothesis regarding the role of openness in moderating emotional responses to the experimental conditions. Although openness has been linked to traits such as curiosity, imagination, and receptivity to new experiences, prior research has provided limited and inconsistent evidence concerning its influence on emotional reactivity in response to distressing stimuli. As a result, openness was included in our analyses for exploratory purposes.

**Figure 1:**
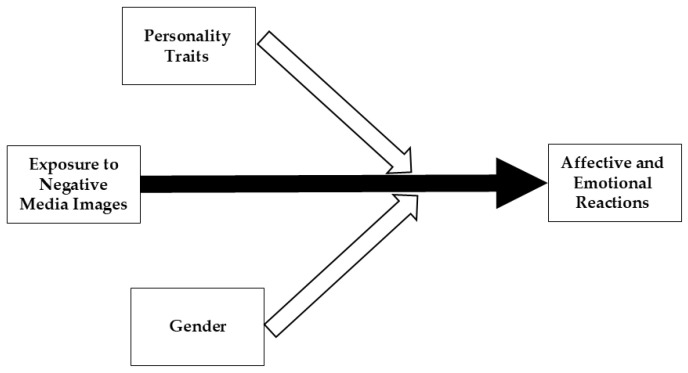
The proposed moderating model depicts the relationship between exposure to negative media images of the 7 October 2023 attacks and affective and emotional responses, moderated by personality traits and gender. The solid black arrow illustrates the direct effect of exposure to negative media images on affective and emotional reactions, whereas the white hollow arrow denotes the moderating influences of personality traits and gender on these associations.

Similarly, we did not have clear a priori predictions about the moderating role that gender may play in our analyses. However, given the well-documented gender differences in emotional processing and expression—such as women’s generally greater emotional sensitivity and empathy (e.g., [62,63])—we included gender as a potential moderator in our analyses to explore whether the associations between personality traits and emotional responses to the different image types varied by gender. Figure 1 illustrates the proposed model examined in this study, highlighting how exposure to negative media images influences emotional and affective responses and identifying the moderating factors involved.

## 2. Materials and Methods

### 2.1. Participants and Procedure

The study included 932 Israeli participants recruited through ‘iPanel,’ a reputable local online panel where individuals register to take part in surveys in exchange for monetary compensation. For their participation, they received 10 ILS (approximately 2.5 USD). The data collection was conducted on a secure online platform, where after providing informed consent, participants completed a series of questionnaires. They were informed that participation was voluntary, that they could withdraw at any time, and that no personally identifiable data would be collected. The study was approved by the Ethics Committee of the Jerusalem Multidisciplinary College (IRB protocol #631). Given the urgency and sensitive nature of the topic, we recruited a convenience sample through iPanel, a well-established online research panel in Israel. This approach enabled the rapid collection of data from individuals directly affected by the attacks. However, the use of convenience sampling introduces the potential for selection bias, as individuals with stronger opinions or greater personal relevance to the topic may have been more likely to participate. It is also possible that volunteers differed systematically from non-volunteers—for example, those higher in neuroticism or those more affected by the attacks may have been more motivated to take part—potentially influencing the representativeness of the sample. Nonetheless, under the circumstances, this method provided timely and valuable insight into the immediate psychological impact of the events.

To ensure data integrity, responses from 106 individuals were excluded based on pre-established criteria. Specifically, participants were excluded for being identified as univariate outliers on one or more study variables (*n* = 44), exhibiting inconsistent response patterns as indicated by high inter-item standard deviation (*n* = 53), or showing invariant response behavior (i.e., “straightlining”) as detected via long-string analysis (*n* = 9). The final sample consisted of 826 participants (423 women, 403 men). The average age was 45.26 years (*SD* = 13.99; range = 18 to 77 years). Marital status was distributed as: 63% married, 14% single, 6% dating, 6% cohabiting, and 12% separated, divorced, or widowed. Participants reported having between 0 and 9 children.

Regarding geographic distribution, participants resided in the following regions: North (26%), South (8%), Central (46%), Jerusalem and surrounding areas (9%), and the Coastal Plain (11%). Religiosity levels were categorized as follows: 60% as secular, 16% as traditional, 11% as religious, and 13% as ultra-Orthodox. Educational attainment was distributed as follows: 40% had completed secondary education, 36% held a bachelor’s degree, 21% possessed a master’s degree or higher, and 3% held a Ph.D.

Employment status showed that 80% of participants were employed, whereas monthly household income was reported relative to the national workforce average: below average by 35%, at the average by 29%, and above average by 36%. Participants also provided information on their political stance, rated on a scale from 1 (left-wing) to 7 (right-wing). The majority of the sample (73%) scored above 4 on this scale, indicating a right-wing orientation (*M* = 4.92, *SD* = 1.49), which reflects the general political landscape in Israel.

Participants reported their exposure to traumatic events related to the October 7th attacks through various channels. These included media and social network exposure, knowing someone who was injured, kidnapped, or killed, having a family member affected, personal involvement such as serving as a soldier or attending the Nova festival, experiencing an attack in their village, spending time in a security or shelter room, or being evacuated from their home. Overall, 73% of participants experienced between 1 and 3 such events (*M* = 2.81, *SD* = 1.19). The dataset is publicly accessible via the Open Science Framework (OSF) at: https://osf.io/aw8e2/.

The procedures involved three main stages:

Stage 1 (Pre-manipulation): Participants completed demographic questionnaires, personality assessments, and baseline measures of affective states and emotions.

Stage 2 (Exposure): Participants were randomly assigned to view three images from one of three categories—explicit negative, implicit negative, or positive—selected based on their content features. The images depicted either explicit death and bodies, scenes of devastation without explicit death, or scenes of reconstruction and renewal. After viewing, they moved to the next stage (see Section 2.1.1 below).

Stage 3 (Post-manipulation): Participants re-rated their affective states and emotions following visual exposure.

#### 2.1.1. Image Selection and Categorization

A total of 32 images captured from Israeli media outlets published shortly after the October 7th attacks were considered. All images appeared on news websites and conformed to journalistic standards prevalent in Israel [37]. These images were characterized by a high degree of exposure and emotional impact.

Two expert judges with backgrounds in visual communication independently categorized these images into three groups: explicit negative (images of covered bodies or deceased individuals), implicit negative (images showing destruction and devastation without explicit death), and positive (images illustrating reconstruction and renewal in the aftermath of terrorism). The judges’ categorizations were highly consistent, as indicated by Kappa statistics: overall Kappa = 0.898 (Standard Error = 0.131, Z = 6.853, *p* < 0.001, 95% Confidence Interval [0.641, 1.155]). Individual category agreements were similarly high (Explicit negative: Kappa = 0.929; Implicit negative: Kappa = 0.875; Positive: Kappa = 0.890), each with significant *p*-values and narrow confidence intervals, supporting the reliability of the categorization process.

From the 32 images, three images per category with unanimous consensus were randomly selected for the experiment. Participants were randomly assigned to view one of the three image categories, with exposure to three images from that category. Each participant’s responses were measured before and after looking at all three images the image exposure to assess changes in affective states and emotions. Images by Category are publicly accessible via the Open Science Framework (OSF) at: https://osf.io/vh7s6/files/osfstorage/6870284607c5d87b293eef6c

A post hoc power analysis, conducted using the observed effect sizes for the main effects of condition on anxiety, demonstrated that our sample size afforded sufficient statistical power (>0.90) to detect significant effects.

#### 2.1.2. Experimental Design

This study employed a mixed factorial design comprising the following:

A within-subjects factor: Time of measurement (pre- and post-exposure); a between-subjects experimental condition factor: Category of images (explicit negative, implicit negative, positive).

### 2.2. Measures

#### 2.2.1. Demographic Questionnaire

Participants responded to a comprehensive demographic questionnaire covering age, gender, education level, region of residence, religiosity, monthly income, occupational status, marital status, number of children, political stance, and levels of exposure to October 7th-related events. This allowed for detailed characterization of the sample and potential control variables in analyses.

#### 2.2.2. Personality

The Big Five Inventory-2 (BFI-2) is a well-validated self-report questionnaire designed to assess the Five-Factor Model of personality, encompassing five broad domains: Extraversion (α = 0.83), Agreeableness (α = 0.79), Conscientiousness (α = 0.88), Neuroticism (α = 0.89), and Openness (α = 0.79). The BFI-2 consists of 60 items rated on a 5-point Likert scale (from 1 = strongly disagree to 5 = strongly agree). The BFI-2 has demonstrated strong reliability and validity across diverse populations, with high internal consistency reported in prior research [64].

#### 2.2.3. Affect and Emotions Measures

*Pre- and Post-Manipulation Specific Emotions and Affective States:*

*Specific Emotions*: The Discrete Emotions Questionnaire (DEQ) [65] was used to measure the intensity and frequency of the following specific emotions: anger (α_Time1_ = 0.87; α_Time2_ = 0.94), disgust (α_Time1_ = 0.82; α_Time2_ = 0.88), fear (α_Time1_ = 0.90; α_Time2_ = 0.95), anxiety (α_Time1_ = 0.80; α_Time2_ = 0.90), sadness (α_Time1_ = 0.80; α_Time2_ = 0.79), desire (α_Time1_ = 0.62; α_Time2_ = 0.60), relaxation (α_Time1_ = 0.91; α_Time2_ = 0.91), and happiness (α_Time1_ = 0.86; α_Time2_ = 0.89). Participants rated their emotional experiences on Likert scales (from 1 = not at all to 7 = an extreme amount). The DEQ’s subscales have exhibited high internal consistency, with Cronbach’s alpha coefficients exceeding 0.85 [65], indicating excellent reliability and construct validity.

*Affective States*: The Affect Grid [66] was employed to assess participants’ current affective states along two core dimensions: valence (pleasure–displeasure) and arousal (high–low). Participants indicated their emotional state by positioning themselves within a 9 × 9 grid, with the *x*-axis representing valence and the *y*-axis representing arousal. The Affect Grid is scored by interpreting the placement of a mark on a 9 × 9 grid, where one axis represents pleasure–displeasure (horizontal) and the other represents arousal–sleepiness (vertical). The grid is structured with the pleasure dimension ranging from −4 (unpleasant) to +4 (pleasant), and the arousal dimension ranging from −4 (sleepy) to +4 (aroused). The final score is a pair of numbers indicating the position on these two dimensions. The Affect Grid is valued for its simplicity, rapid administration, and demonstrated reliability and validity across different populations [66].

### 2.3. Statistical Analyses

First, descriptive statistics (frequency distributions, means, and standard deviations) were calculated for participants’ background and sociodemographic characteristics. Differences among participants assigned to the three conditions were examined to identify any potential covariates for subsequent analyses. Then, we analyzed the Pearson product–moment correlation coefficients that personality traits had with specific emotions and affect states. This was followed by a series of ten 2 (Time: Pre-Exposure vs. Post-Exposure) × 3 (Condition: Explicit Negative vs. Implicit Negative vs. Positive) mixed-model ANOVAs that were conducted to examine changes in specific emotions and affective states following exposure to the experimental manipulation. Finally, we conducted a series of hierarchical moderated multiple regression analyses to examine whether personality traits and gender moderated participants’ emotional and affective responses following the experimental manipulation. In Step 1 of each analysis, we entered two dummy-coded variables representing Condition, with the implicit negative condition serving as the reference group given that it was often associated with the most intense post-exposure negative emotions. This step also included the Big Five personality traits, gender, and the pre-exposure score for the specific emotional outcome being analyzed (e.g., pre-exposure anger was included when post-exposure anger was the outcome). Step 2 added all relevant two-way interaction terms, and Step 3 included the three-way interactions among condition, personality traits, and gender. All continuous predictor variables centered for the purpose of testing interactions. Simple slopes tests were performed to clarify any interactions that emerged, using values one standard deviation above and below the mean to represent high or low levels of the continuous variables.

## 3. Results

### 3.1. Background and Sociodemographic Variables

Table 1 presents descriptive statistics for participants’ background and sociodemographic characteristics. Comparisons across exposure groups revealed no statistically significant differences for most demographic variables. Specifically, no significant group differences emerged for political affiliation or cumulative exposure to the events of October 7th. Although a statistically significant difference in religiosity was initially observed—with the majority of participants in each group identifying as secular—this effect did not remain significant after correcting the *p*-value for multiple comparisons. As a result, the demographic variables shown in Table 1 were not included in subsequent analyses, in order to maintain clarity and focus.

### 3.2. Mixed-Model ANOVAs

As summarized in Table 2, there were consistent main effects of Time, such that negative emotions (i.e., anger, disgust, fear, anxiety, and sadness) increased and positive emotions (i.e., desire, relaxation, and happiness) decreased from pre-exposure to post-exposure. In addition, participants reported more negative valence and higher arousal for their affective states following exposure. Effects involving Condition revealed a consistent pattern in which the positive condition elicited lower levels of negative emotions and higher levels of positive emotions compared to both the explicit and implicit negative conditions. In some cases, the implicit negative condition was associated with greater increases in negative emotion than the explicit negative condition (i.e., anger, anxiety, and sadness), though for other emotions, these two conditions did not differ significantly (i.e., disgust and fear).

### 3.3. Zero-Order Correlations

Table 3 presents the correlation coefficients between personality traits and specific emotions and affective states both before and after exposure to experimental manipulation. The pre-exposure pattern revealed that extraversion, agreeableness, and conscientiousness were negatively correlated with negative emotions, whereas neuroticism was positively correlated with these emotions. Openness also tended to show negative associations with negative emotions, but these correlations were generally weaker and less consistent than those observed for the other traits.

In contrast, extraversion, agreeableness, conscientiousness, and openness were positively associated with pre-exposure positive emotions, valence, and arousal. Neuroticism, by comparison, was negatively correlated with these positive affective states and valence, although it showed a positive association with arousal.

Following the experimental manipulation, personality traits showed relatively few significant associations with post-exposure emotions and affective states across the full sample, likely due to the overriding influence of the experimental conditions. One notable exception was neuroticism, which remained positively correlated with each of the post-exposure negative emotions and negatively correlated with relaxation, happiness, and arousal.

### 3.4. Hierarchical Moderated Multiple Regression Results

#### 3.4.1. Anger

Pre-exposure anger was positively associated with post-exposure anger (β = 0.27, t = 8.38, *p* < 0.001). Participants in the implicit negative condition reported significantly higher levels of post-exposure anger than those in either the positive condition (β = −0.68, t = −22.56, *p* < 0.001) or the explicit negative condition (β = −0.15, t = −4.82, *p* < 0.001). Among the Big Five personality traits, extraversion (β = 0.07, t = 2.30, *p* = 0.022) and agreeableness (β = 0.10, t = 3.16, *p* = 0.002) were both positively associated with post-exposure anger. No other significant main effects were observed.

**Figure 2:**
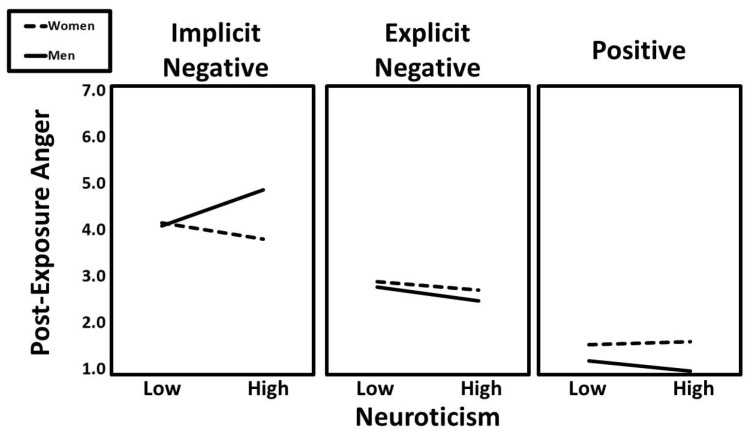
The predicted values for post-exposure anger illustrating the interaction that neuroticism had with gender and experimental condition.

Gender moderated the effects of both dummy-coded condition contrasts: implicit negative versus positive (β = −0.14, t = −3.52, *p* < 0.001) and implicit negative versus explicit negative (β = −0.09, t = −2.32, *p* = 0.021). However, these moderation effects were further qualified by three significant three-way interactions. The first was a significant neuroticism × condition (implicit vs. positive) × gender interaction (β = −0.10, t = −2.14, *p* = 0.032). Predicted values for this interaction are presented in Figure 2. Follow-up simple slopes analyses indicated that the positive association between neuroticism and post-exposure anger was particularly strong for men in the implicit negative condition (β = 0.31, t = 2.95, *p* = 0.004).

**Figure 3:**
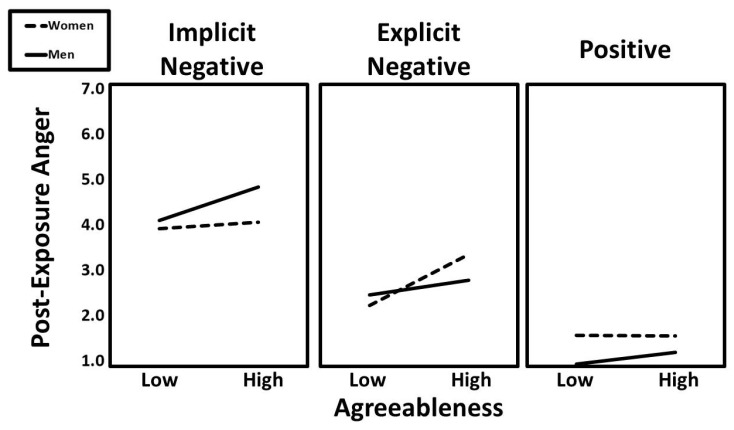
The predicted values for post-exposure anger illustrating the interaction that agreeableness had with gender and experimental condition.

The second three-way interaction was agreeableness × condition (implicit vs. explicit) × gender (β = −0.10, t = −2.35, *p* = 0.019). Predicted values for this interaction are presented in Figure 3. Follow-up simple slopes analyses indicated that the positive association between agreeableness and post-exposure anger was particularly strong for men in the implicit negative condition (β = 0.23, t = 2.38, *p* = 0.019) and women in the explicit negative condition (β = 0.31, t = 3.34, *p* < 0.001).

**Figure 4:**
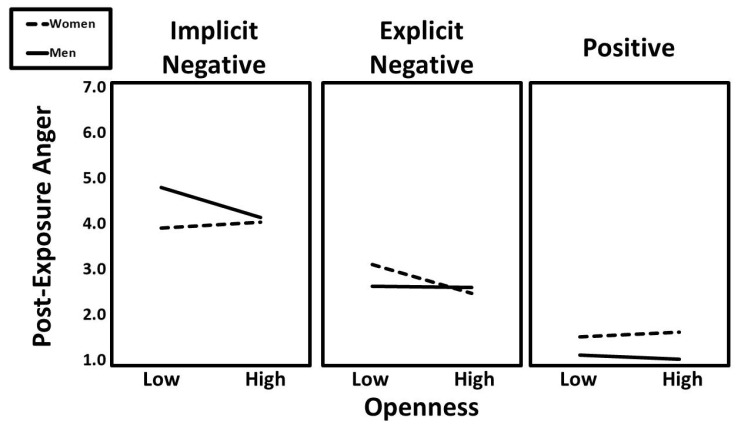
The predicted values for post-exposure anger illustrating the interaction that openness had with gender and experimental condition.

The final three-way interaction was openness × condition (implicit vs. explicit) × gender (β = 0.10, t = 2.40, *p* = 0.017). Predicted values for this interaction are presented in Figure 4. Follow-up simple slopes analyses indicated that the negative association between openness and post-exposure anger was particularly strong for men in the implicit negative condition (β = −0.21, t = −2.28, *p* = 0.024) and women in the explicit negative condition (β = −0.17, t = −2.03, *p* = 0.044).

#### 3.4.2. Disgust

Pre-exposure disgust was positively associated with post-exposure disgust (β = 0.22, t = 7.25, *p* < 0.001). Participants in the implicit negative condition reported significantly higher levels of post-exposure disgust than those in the positive condition (β = −0.61, t = −18.99, *p* < 0.001), but not significantly more than those in the explicit negative condition (β = −0.06, t = −1.88, *p* = 0.061). Among the Big Five personality traits, extraversion (β = 0.07, t = 2.21, *p* = 0.028) and agreeableness (β = 0.10, t = 3.06, *p* = 0.002) were positively associated with post-exposure disgust, whereas openness was negatively associated (β = −0.09, t = −2.90, *p* = 0.004). No other significant main effects were observed.

**Figure 5:**
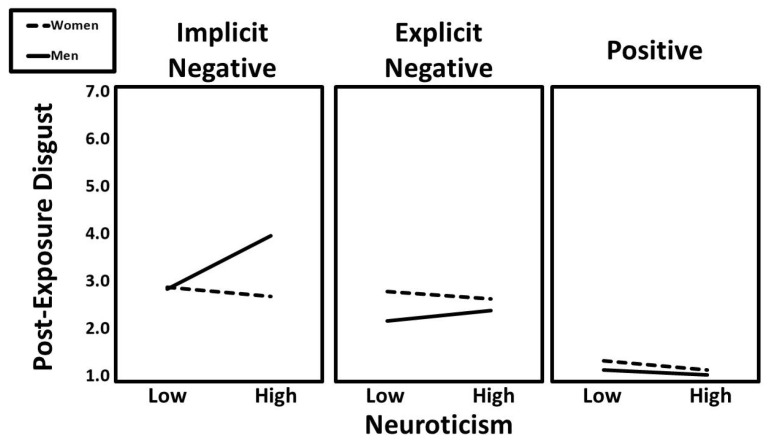
The predicted values for post-exposure disgust illustrating the interaction that neuroticism had with gender and experimental condition.

Several two-way interactions significantly predicted post-exposure disgust, including neuroticism × condition (implicit vs. positive; β = −0.11, t = −2.35, *p* = 0.019), neuroticism × gender (β = 0.08, t = 2.34, *p* = 0.019), and condition (implicit vs. positive) × gender (β = −0.11, t = −2.65, *p* = 0.008). However, all three interactions were qualified by a significant three-way interaction of neuroticism × condition (implicit vs. positive) × gender (β = −0.10, t = −2.14, *p* = 0.033). Predicted values for this interaction are shown in Figure 5. Follow-up simple slopes analyses revealed that the positive association between neuroticism and post-exposure disgust was especially pronounced for men in the implicit negative condition (β = 0.34, t = 3.40, *p* < 0.001).

#### 3.4.3. Fear

Pre-exposure fear was positively associated with post-exposure fear (β = 0.43, t = 13.17, *p* < 0.001). Participants in the implicit negative condition reported significantly higher levels of post-exposure fear than those in either the positive condition (β = −0.53, t = −17.04, *p* < 0.001) or the explicit negative condition (β = −0.12, t = −3.94, *p* < 0.001). Among the Big Five personality traits, agreeableness was positively associated with post-exposure fear (β = 0.11, t = 3.42, *p* < 0.001). No other significant main effects emerged.

**Figure 6:**
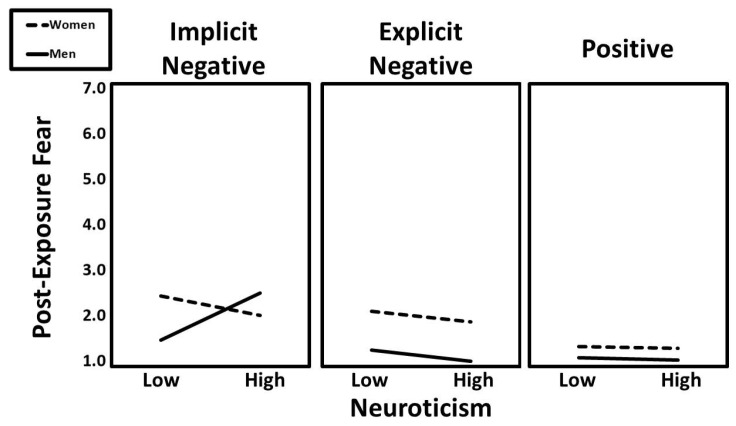
The predicted values for post-exposure fear illustrating the interaction that neuroticism had with gender and experimental condition.

A significant two-way interaction between neuroticism and condition (implicit vs. positive) predicted post-exposure fear (β = −0.12, t = −2.46, *p* = 0.014), but this effect was further qualified by a significant three-way interaction involving neuroticism, condition (implicit vs. positive), and gender (β = −0.11, t = −2.28, *p* = 0.023). Additionally, a separate three-way interaction of neuroticism × condition (implicit vs. explicit) × gender was also significant (β = −0.11, t = −2.43, *p* = 0.015). Predicted values for this interaction are presented in Figure 6. Follow-up simple slopes analyses indicated that the positive association between neuroticism and post-exposure fear was especially pronounced for men in the implicit negative condition (β = 0.24, t = 2.50, *p* = 0.014).

#### 3.4.4. Anxiety

Pre-exposure anxiety was positively associated with post-exposure anxiety (β = 0.40, t = 11.15, *p* < 0.001). Participants in the implicit negative condition reported significantly higher levels of post-exposure anxiety than those in either the positive condition (β = −0.56, t = −18.20, *p* < 0.001) or the explicit negative condition (β = −0.12, t = −3.70, *p* < 0.001). Among the personality traits, both extraversion (β = 0.08, t = 2.40, *p* = 0.017) and agreeableness (β = 0.11, t = 3.58, *p* < 0.001) were positively associated with post-exposure anxiety. No other significant main effects were observed.

**Figure 7:**
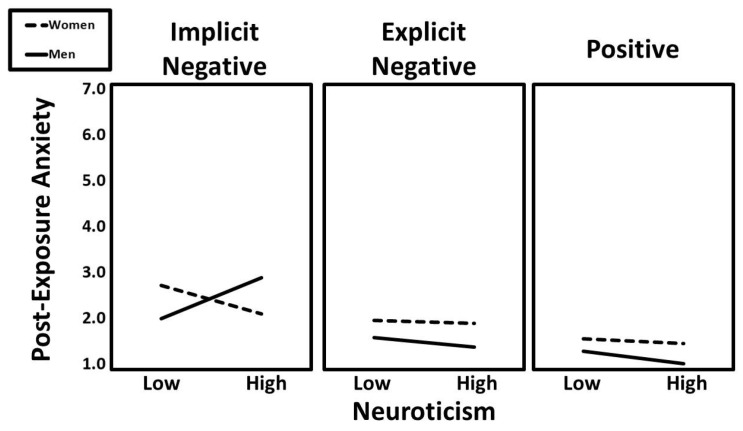
The predicted values for post-exposure anxiety illustrating the interaction that neuroticism had with gender and experimental condition.

A significant two-way interaction emerged between neuroticism and condition (implicit vs. positive; β = −0.11, t = −2.37, *p* = 0.018), but this effect was further qualified by a significant three-way interaction involving neuroticism, condition (implicit vs. positive), and gender (β = −0.13, t = −2.79, *p* = 0.005). An additional three-way interaction of neuroticism × condition (implicit vs. explicit) × gender also significantly predicted post-exposure anxiety (β = −0.13, t = −2.83, *p* = 0.005). Predicted values for this interaction are shown in Figure 7. Follow-up simple slopes analyses revealed that the positive association between neuroticism and post-exposure anxiety was especially strong for men in the implicit negative condition (β = 0.30, t = 2.77, *p* = 0.007).

#### 3.4.5. Sadness

Results for sadness indicated that pre-exposure sadness was positively associated with post-exposure sadness (β = 0.34, t = 10.04, *p* < 0.001). Participants in the implicit negative condition reported significantly higher levels of post-exposure sadness than those in either the positive condition (β = −0.62, t = −20.12, *p* < 0.001) or the explicit negative condition (β = −0.12, t = −3.95, *p* < 0.001). Among the personality traits, extraversion (β = 0.07, t = 2.21, *p* = 0.027) was positively associated with post-exposure sadness. No other significant main effects emerged.

**Figure 8:**
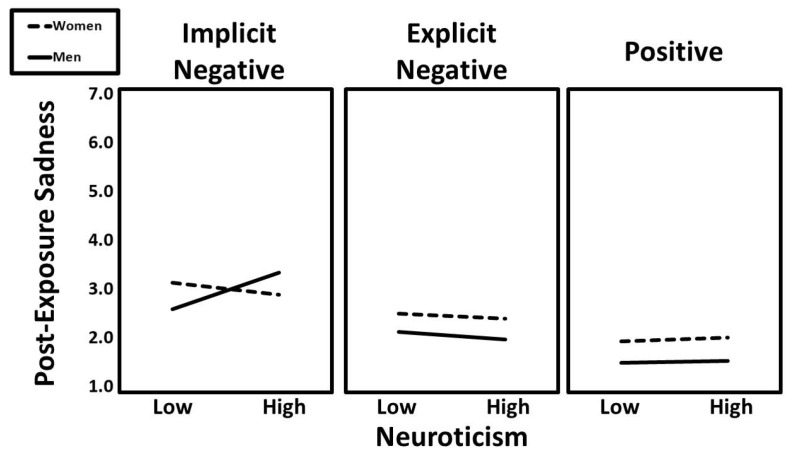
The predicted values for post-exposure sadness illustrating the interaction that neuroticism had with gender and experimental condition.

The two-way interactions of neuroticism × condition (implicit vs. positive; β = −0.11, t = −2.34, *p* = 0.026) and condition (implicit vs. positive) × gender (β = −0.08, t = −2.08, *p* = 0.038) were significant predictors of post-exposure sadness, but they were qualified by the three-way interaction of neuroticism × condition (implicit vs. positive) × gender (β = −0.11, t = −2.34, *p* = 0.026). In addition, the three-way interaction of neuroticism × condition (implicit vs. explicit) × gender was a significant predictor of post-exposure sadness (β = −0.10, t = −2.26, *p* = 0.024). Predicted values for this interaction are presented in Figure 8. Follow-up simple slopes analyses indicated that the positive association between neuroticism and post-exposure sadness was especially pronounced for men in the implicit negative condition (β = 0.29, t = 2.99, *p* = 0.003).

#### 3.4.6. Desire

Pre-exposure desire was positively associated with post-exposure desire (β = 0.33, t = 9.50, *p* < 0.001). Participants in the implicit negative condition reported significantly higher levels of post-exposure desire than those in the explicit negative condition (β = −0.12, t = −3.06, *p* = 0.002), but not significantly more than those in the positive condition (β = −0.62, t = −20.12, *p* < 0.001). Among the personality traits, extraversion was positively associated with post-exposure desire (β = 0.08, t = 2.05, *p* = 0.041), whereas openness was negatively associated (β = −0.10, t = −2.74, *p* = 0.006). No other significant main effects or interactions were observed.

#### 3.4.7. Relaxation

Pre-exposure relaxation was positively associated with post-exposure relaxation (β = 0.20, t = 5.06, *p* < 0.001). Participants in the implicit negative condition reported significantly lower levels of post-exposure relaxation than those in the positive condition (β = 0.51, t = 14.94, *p* < 0.001), but not significantly lower than those in the explicit negative condition (β = 0.06, t = 1.81, *p* = 0.070). Among the personality traits, openness was negatively associated with post-exposure relaxation (β = −0.08, t = −2.34, *p* = 0.019). No other significant main effects or interactions were observed.

#### 3.4.8. Happiness

Pre-exposure happiness was positively associated with post-exposure happiness (β = 0.19, t = 5.79, *p* < 0.001). Participants in the implicit negative condition reported significantly lower levels of post-exposure happiness than those in the positive condition (β = 0.62, t = 20.30, *p* < 0.001), but they did not differ significantly from those in the explicit negative condition (β = 0.00, t = 0.05, *p* = 0.958). Among the personality traits, openness was negatively associated with post-exposure happiness (β = −0.10, t = −3.41, *p* < 0.001). No other significant main effects or interactions were observed.

#### 3.4.9. Valence

Pre-exposure valence was positively associated with post-exposure valence (β = 0.12, t = 2.87, *p* = 0.004). However, participants in the implicit negative condition did not significantly differ in post-exposure valence from those in either the positive condition (β = 0.03, t = 0.79, *p* = 0.428) or the explicit negative condition (β = −0.05, t = −1.17, *p* = 0.242). Among the personality traits, agreeableness (β = −0.21, t = −4.51, *p* < 0.001) and neuroticism (β = −0.21, t = −4.57, *p* < 0.001) were both negatively associated with post-exposure valence. No other significant main effects or interactions were observed.

#### 3.4.10. Arousal

Participants in the implicit negative condition reported significantly higher levels of post-exposure arousal than those in the positive condition (β = −0.76, t = −29.00, *p* < 0.001), but they did not differ significantly from those in the explicit negative condition (β = −0.01, t = −0.38, *p* = 0.707). Among the personality traits, both neuroticism (β = 0.07, t = 2.58, *p* = 0.010) and openness (β = 0.07, t = 2.65, *p* = 0.008) were positively associated with post-exposure arousal. No other significant main effects or interactions were observed.

## 4. Discussion

The present study provides important insights into the psychological burden of terrorism and highlights how individual differences shape emotional responses to terror-related media content. As a product of transdisciplinary research, these findings carry significant implications for mental health promotion and public health strategies explicitly aimed at reducing the adverse psychological effects of terrorism on well-being and quality of life. By identifying key personality traits that heighten vulnerability, this work can inform the development of targeted interventions and support systems to strengthen resilience in at-risk populations.

The present study aimed to examine the emotional impact of exposure to media images depicting the October 7th terror attacks, with particular attention to the moderating role of personality traits. Our findings offer several important insights into how individuals emotionally respond to such distressing visual content related to terrorism.

As assumed, exposure to both explicit and implicit negative images significantly increased negative emotions (i.e., anger, disgust, fear, anxiety, and sadness) while decreasing positive emotions (i.e., desire, relaxation, and happiness). These findings reinforce the potent influence of visual media in eliciting emotional distress on people that sympathize and identify with the victims in the context of terrorism, as well as align with previous research on the psychological effects of exposure to traumatic imagery [23,24,25]. The findings further indicate that implicit representation of violet death can arouse stronger reaction than explicit images. Although journalists have ethical groundings to refrain from explicit, gruesome death imagery in the aftermath of terrorism, they should also pay attention to potential distress as a result of exposure to implicit death imagery. Furthermore, digital platforms also need to be mindful of potential abuse of their infrastructure either by the terrorists themselves or by layperson unaware of ethical guidelines.

In support of our first hypothesis (H1), neuroticism emerged as a key moderator of emotional response, particularly among men exposed to implicitly negative images. Individuals high in neuroticism exhibited greater emotional reactivity, consistent with previous findings linking this trait to heightened sensitivity to threat and negative affectivity. These findings underscore the importance of incorporating individual psychological vulnerabilities—a key biological and psychological determinant of health—into public health responses to terrorism. The gender-specific pattern observed suggests that men high in neuroticism may be particularly vulnerable to distress from subtle or ambiguous negative stimuli, a possibility that warrants further exploration in future research.

Interestingly, agreeableness also moderated emotional responses. Higher levels of agreeableness were associated with increased anger in certain conditions—specifically, among men exposed to implicit negative images and women exposed to explicit negative images. Although this finding may appear counterintuitive, it may reflect empathic distress or moral outrage in response to visual depictions of violence and suffering. That is, individuals high in agreeableness may respond with anger when confronted with perceived injustice or human suffering.

Contrary to our expectations, conscientiousness and extraversion did not consistently moderate emotional responses. These null findings may reflect the specific characteristics of the visual stimuli or the complex interactions between dispositional and situational factors. It is also possible that the influence of these traits on emotional processing is more subtle and would require alternative methodological approaches to detect.

Openness to experience, however, was negatively associated with post-exposure anger in certain contexts. This suggests that individuals high in openness may be more capable of emotionally regulating their responses to distressing content or may interpret such images through a more reflective or detached lens, reducing emotional reactivity.

These findings have important implications for understanding the role of media in shaping emotional responses during times of conflict. On news outlets, journalists have well-established norms and practices designed to mitigate the harmful potential of violent reality [37]. But in an era of constant digital connectivity, individuals may be exposed to graphic or disturbing content online. In some cases, like centralized platforms, the platforms attempt to reinforce their community standards and remove harmful content, but in decentralized platforms, such content can travel rapidly and freely [44]. Gaining insight into how such exposure affects people differently is crucial for guiding ethical media practices and informing public health strategies.

Our finding that implicit negative imagery had a stronger negative impact on individuals high in neuroticism highlights the need for media outlets to consider the psychological consequences of *how* violence is depicted. Although implicit negative imagery may appear less graphic than explicit depictions, it can be particularly distressing to vulnerable individuals. Accordingly, while accurate and transparent reporting remains essential, media organizations should consider implementing safeguards such as trigger warnings, blurring techniques, or user-controlled display options to help viewers make informed decisions about exposure. This is especially important on platforms frequented by individuals more prone to emotional distress, such as those high in neuroticism.

The capacity of textual framing to alter the emotional impact of disturbing visuals [23,25] further underscores the ethical responsibility of journalism to frame and mediate violent realities carefully. Beyond content warnings, media outlets could offer adjustable display settings tailored to viewer sensitivity or provide contextual information that guides emotional processing. Our findings that agreeableness was associated with heightened anger in response to explicit images also suggest that viewing such material can carry a disproportionate emotional toll for some individuals. Meanwhile, the rise of decentralized and algorithm-driven media platforms, which often circulate visual content without journalistic mediation or contextual framing [6], poses additional challenges. These platforms should therefore develop mechanisms—such as content filters, opt-in warnings, or AI-based moderation—to mitigate potential harm.

Our results further indicate that individuals higher in extraversion may exhibit greater resilience to violent imagery. Public health campaigns can capitalize on this by providing coping resources and psychoeducational materials that promote adaptive emotion regulation and offering viewers information so they can make informed decisions about viewership. In times of national trauma or armed conflict, it is also critical that media outlets combat misinformation and avoid sensationalism by verifying information from multiple reliable sources, clearly distinguishing between facts and opinions, and avoiding emotionally charged language or framing. Responsible, evidence-based journalism can help reduce emotional distress and prevent polarization during crises. Based on the present findings, such communication strategies may be particularly beneficial when targeted toward individuals with higher agreeableness, who may be especially reactive to emotionally charged content.

Beyond the realm of journalism, these findings carry broader implications for mental health professionals, educators, and policymakers. Our findings indicate that people high in neuroticism have stronger reactions, so clinicians should be aware of the potential emotional toll of media exposure on their clients, especially those with elevated neuroticism or a history of trauma. Screening for media-related distress may be a valuable component of trauma-informed care to determine the effect of personality on how they regulate their responses. Educational initiatives can play a key role by fostering media literacy and enhancing individuals’ ability to critically engage with emotionally charged content but specifically focus that content to promote better cognitive understanding among individuals who have high openness to understand the tools and resources at their disposal. Encouraging open dialog and social support around reactions to traumatic media exposure may also promote resilience and reduce psychological burden but should also focus on methods that assist extraverted people who report less impact from negative imagery. From a policy perspective, lawmakers may need to consider regulations that address the dissemination of violent or harmful content online. Such efforts must balance the protection of public mental health with respect for freedom of expression such as ensuring there are resources available to all and to ensure responsible and sensitive use by the media.

In light of these findings, it is essential for mental health professionals, educators, and policymakers to recognize the potential emotional toll that media exposure to terrorism can impose and to translate this knowledge into effective health promotion practices. Key strategies include promoting media literacy, fostering open dialog about reactions to traumatic events, and ensuring access to resources that support coping with emotional distress. Such efforts are vital for building resilience, enhancing well-being, and reducing the psychological burden of terrorism. Future research should prioritize the development and evaluation of interventions that draw on these insights to strengthen mental health outcomes and improve quality of life in affected communities, with a sustained focus on health promotion.

The current study has several limitations that should be considered when interpreting the findings. First, the sample consisted primarily of Israeli Jews, which were the direct victim of the October 7th attacks. While most other studies on the impact of visual imagery on audiences are also confined to a specific cultural context, this may limit the generalizability of the results to other cultural or demographic groups. Future research should therefore include more diverse populations—such as Ukrainians who are experiencing psychological trauma due to ongoing conflict and Palestinians who are also affected by regional violence—to enhance external validity. Examining whether similar patterns of personality-driven emotional responses emerge across different cultural contexts would provide valuable insights into the cross-cultural applicability of these findings and clarify whether the observed relationships between media exposure, personality traits, and emotional responses generalize to other populations affected by terrorism worldwide.

Second, the reliance on self-report measures introduces potential biases, including social desirability and inaccurate reporting. Participants may underreport or overreport their emotional reactions depending on social norms or other factors. To address this limitation, future studies should incorporate objective measures of emotional response, such as physiological indicators (e.g., heart rate, skin conductance) or behavioral observations, to provide a more nuanced and reliable assessment of participants’ reactions to terrorism-related media content.

Third, the cross-sectional design of the study precludes causal inferences. Longitudinal research would allow for a more precise understanding of how repeated exposure to terrorism-related media influences emotional and psychological outcomes over time and how personality traits may shape or moderate these trajectories. Such designs would also allow for a more precise understanding of the long-term psychological consequences of ongoing exposure to violent or distressing media content.

Finally, the absence of a control group is another limitation. Although we sought to minimize distress by ensuring that all participants viewed some form of visual stimuli related to the events and that measures were tested before exposure to any stimuli, the lack of a true control condition makes it difficult to isolate the specific effects of the experimental manipulation. We determined that asking participants from this population to recall their emotional state without any contextual stimulus could elevate anxiety to a degree that might compromise the reliability of their responses. Future research should consider incorporating an ethically appropriate control condition—such as participants exposed to neutral imagery—to strengthen causal interpretations. Furthermore, still images are often uniquely perceived as capable of capturing a moment in time that can become iconic, especially in the aftermath of wars and terrorism. And yet, since this study focused exclusively on still photographs, future research should examine other media formats, including videos, social media posts, and user-generated content. These formats may elicit different emotional responses and vary in accessibility, realism, and psychological impact, offering a more comprehensive understanding of media effects in contexts of terrorism and collective trauma. Despite these limitations, the present study contributes important insights into the complex interplay between media exposure, personality, and emotional response. These findings underscore the need for future research to replicate and extend this work in more diverse populations, to incorporate objective and longitudinal methodologies, and to identify practical strategies for mitigating the harmful effects of repeated exposure to terrorism-related media content.

### Implications for Health Promotion and Public Health Practice

The findings of this study carry important implications for health promotion and public health practice, particularly in the context of terrorism and other traumatic events.

First, our results underscore the need to recognize individual psychological vulnerabilities when designing public health responses to terrorism. Identifying neuroticism as a key moderator of emotional reactions highlights the importance of tailored interventions for at-risk individuals. Mental health professionals and public health practitioners can use this knowledge to develop targeted support programs that promote resilience and mitigate the adverse effects of media exposure.

Second, our findings provide direct guidance for responsible media practices. By understanding how different types of imagery influence emotional responses, media outlets can adopt ethical guidelines that minimize harm and protect public well-being. This includes limiting gratuitous or graphic content, providing clear warnings for potentially disturbing material, and offering resources to help audiences cope with distress. Media literacy campaigns can further strengthen resilience by fostering critical engagement with media and encouraging individuals who regulate their exposure during crises.

Third, these insights may inform the development of educational initiatives aimed at improving media literacy and equipping individuals with coping strategies. By raising awareness about the psychological impact of media exposure, such programs can empower people to make informed decisions about their media consumption and protect themselves from emotional harm, while also fostering resilience in the face of adversity.

Beyond terrorism, these findings can be applied to other forms of traumatic events and media exposure. Identifying psychological factors that shape emotional responses can guide the design of interventions and policies that support mental health and resilience across diverse contexts. Future research should evaluate the effectiveness of interventions such as cognitive-behavioral therapy, mindfulness-based programs, and community-based support, as well as explore the best strategies for disseminating responsible media practices and promoting media literacy at scale.

By translating these findings into practical interventions, policies, and educational initiatives, public health systems can better promote resilience, enhance well-being, and reduce the psychological burden of terrorism and related traumatic events—advancing core goals of health promotion.

## 5. Conclusions

In conclusion, this study highlights the substantial psychological impact of exposure to terror-related media images—whether explicit negative, implicit negative, or positive—and underscores the crucial role that personality traits play in shaping individual emotional responses. These findings point to the urgent need for responsible media practices, tailored mental health interventions, and educational efforts aimed at reducing the potential harm caused by distressing visual content—particularly in contexts of war and conflict. Moving forward, a coordinated and multidisciplinary approach involving media professionals, mental health practitioners, educators, and policymakers will be essential for promoting resilience and safeguarding psychological well-being amid ongoing global challenges. By identifying the personality traits that heighten vulnerability to negative emotional reactions, interventions can be designed to deliver tailored support and coping strategies, thereby strengthening resilience and improving mental health outcomes at the population level.

## Figures and Tables

**Table 1:** Sociodemographic and background information.

	Total Sample (*N* = 826)	Explicit Negative (*n* = 279)	Implicit Negative (*n* = 269)	Positive (*n* = 278)	Statistic
**Sex**					χ^2^_[df=2]_ = 0.72, *p* > 0.05
*Women*	51.2%	53%	49.4%	51.1%	
*Men*	48.8%	47%	50.6%	48.9%	
**Age**	45.26 (13.99)	44.95 (13.98)	45.10 (14.34)	45.73 (13.69)	F_[df=2, 823]_ = 0.24, *p* > 0.05
**Number of children**	2.77 (1.40)	2.88 (1.53)	2.56 (1.31)	2.83 (1.31)	F_[df=2, 583]_ = 2.86, *p* > 0.05
**Right-wing Political Stance**	4.92 (1.49)	4.96 (1.43)	4.84 (1.52)	4.96 (1.52)	F_[df=2, 823]_ = 0.57, *p* > 0.05
**Cumulative Exposure**	2.81 (1.19)	2.76 (1.19)	2.83 (1.17)	2.85 (1.21)	F_[df=2, 823]_ = 0.41, *p* > 0.05
**Employment Status**					
*Full time*	65.9%	59.5%	65.7%	73.4%	χ^2^_[df=18]_ = 23.83, *p* > 0.05
*Part time*	14.5%	19.4%	14.1%	10.1%	
*On leave*	0.6%	1.1%	0.4%	0.4%	
*Temporarily laid off*	0.1%	0.4%	0.0%	0.0%	
*Unemployed and looking for work*	3.3%	4.7%	2.6%	2.5%	
*Unemployed health-related reason*	1.0%	1.1%	1.1%	0.7%	
*Going to school*	5.7%	5.4%	7.4%	4.3%	
*Home maker*	1.3%	1.8%	1.1%	1.1%	
*Retired*	5.4%	5.4%	6.3%	4.7%	
*Disability payments*	2.2%	1.4%	2.2%	2.9%	
**Religiosity**					χ^2^_[df=6]_ = 14.92, *p* < 0.03
*Secular*	60.0%	53.8%	63.2%	63.3%	
*Traditional*	15.7%	15.8%	17.8%	13.7%	
*Religious*	11.4%	12.5%	8.9%	12.6%	
*Ultra-Orthodox*	12.8%	17.9%	10.0%	10.4%	
**Marital Status**					χ^2^_[df=14]_ = 12.07, *p* > 0.05
*Single*	14.0%	14.7%	14.9%	12.6%	
*Casually dating*	1.6%	1.8%	2.2%	0.7%	
*Seriously dating*	4.2%	3.9%	5.6%	3.2%	
*Cohabiting*	5.7%	4.7%	5.9%	6.5%	
*Married*	62.8%	65.6%	59.5%	63.3%	
*Separated*	0.5%	0.4%	0.0%	1.1%	
*Divorced*	10.7%	8.6%	11.2%	12.2%	
*Widowed*	0.5%	0.4%	0.7%	0.4%	
**Education**					χ^2^_[df=8]_ = 4.66, *p* > 0.05
*High school no Matriculation*	13.1%	12.9%	11.5%	14.7%	
*High school full Matriculation*	27.0%	26.2%	28.6%	26.3%	
*Bachelor’s degree*	36.3%	36.2%	34.9%	37.8%	
*Master’s degree or higher*	20.7%	21.9%	20.8%	19.4%	
*Ph.D.*	2.9%	2.9%	4.1%	1.8%	
**Monthly gross household income**					χ^2^_[df=8]_ = 5.99, *p* > 0.05
*Much higher than* *13,385 NIS*	11.1%	10.0%	11.2%	12.2%	
*Higher than* *13,385 NIS*	24.9%	22.9%	23.0%	28.8%	
*Equals* *13,385 NIS*	28.7%	30.1%	30.9%	25.2%	
*Lower than* *13,385 NIS*	22.2%	24.0%	20.8%	21.6%	
*Much Lower than* *13,385 NIS*	13.1%	12.9%	14.1%	12.2%	
**Place of Residence**					χ^2^_[df=8]_ = 8.34, *p* > 0.05
*Northen*	26.3%	24.4%	29.0%	25.5%	
*Central*	45.5%	44.4%	44.6%	47.5%	
*Coastal Plain*	11.35%	10.0%	10.8%	12.9%	
*Jerusalem Region*	9.3%	12.5%	8.6%	6.8%	
*Southern*	7.6%	8.6%	7.1%	7.2%	

**Table 2:** Comparisons of experimental conditions across pre- and post-exposure.

	Explicit Negative		Implicit Negative		Positive				
	Pre-	Post-	Pre-	Post-	Pre-	Post-	Time	Condition	Time × Condition
Anger	2.56	4.57	2.70	5.21	2.66	2.39	628.30 ***	136.80 ***	230.00 ***
Disgust	2.18	3.74	2.23	3.99	2.22	1.68	302.85 ***	119.74 ***	191.70 ***
Fear	2.23	3.59	2.17	4.00	2.20	1.94	344.51 ***	60.47 ***	143.78 ***
Anxiety	2.83	4.03	2.89	4.47	2.87	2.38	235.19 ***	71.04 ***	162.74 ***
Sadness	2.49	3.86	2.59	4.25	2.54	2.41	491.92 ***	77.82 ***	163.61 ***
Desire	3.83	2.28	3.91	2.60	3.91	2.45	1165.54 ***	3.71 *	2.82
Relaxation	4.28	1.59	4.37	1.47	4.41	2.76	2575.47 ***	53.11 ***	67.02 ***
Happiness	4.69	1.32	4.62	1.32	4.74	3.07	3818.74 ***	121.18 ***	152.65 ***
Valence	−1.22	−1.55	−0.97	−1.29	−1.17	−1.20	6.57 *	2.53	1.22
Arousal	−0.89	3.03	−0.78	3.04	−0.61	−0.90	892.91 ***	241.17 ***	279.59 ***

* *p* < 0.05; *** *p* < 0.001.

**Table 3:** The zero-order correlation coefficients that personality traits had with specific emotions and affective states pre- and post-exposure to the experimental manipulation.

	Extraversion (Pre-/Post-)	Agreeableness (Pre-/Post-)	Conscientiousness (Pre-/Post-)	Neuroticism (Pre-/Post-)	Openness (Pre-/Post-)
Anger	−0.13 ***/0.03	−0.40 ***/0.00	−0.25 ***/0.00	0.54 ***/0.09 **	−0.07 */−0.03
Disgust	−0.14 ***/0.00	−0.35 ***/0.00	−0.28 ***/−0.04	0.38 ***/0.07 *	−0.10 **/−0.08 *
Fear	−0.21 ***/−0.01	−0.23 ***/0.06	−0.30 ***/−0.03	0.57 ***/0.20 ***	−0.13 ***/−0.03
Anxiety	−0.20 ***/0.00	−0.25 ***/0.04	−0.25 ***/−0.01	0.67 ***/0.21 ***	−0.04/−0.02
Sadness	−0.30 ***/−0.03	−0.28 ***/−0.01	−0.32 ***/−0.04	0.60 ***/0.20 ***	−0.06/0.01
Desire	0.29 ***/0.10 **	0.03/−0.03	0.07 */−0.03	−0.09 */0.01	0.21 ***/−0.01
Relaxation	0.24 ***/0.04	0.31 ***/−0.02	0.25 ***/0.02	−0.65 ***/−0.15 ***	0.09 */−0.04
Happiness	0.44 ***/0.04	0.32 ***/0.03	0.29 ***/0.02	−0.52 ***/−0.10 **	0.23 ***/−0.04
Valence	0.34 ***/0.04	0.25 ***/0.17 ***	0.26 ***/0.08 *	−0.56 ***/0.06	0.10 **/0.03
Arousal	0.22 ***/0.05	0.10 **/0.00	0.10 **/0.00	0.15 ***/−0.10 **	0.14 ***/−0.01

* *p* < 0.05; ** *p* < 0.01; *** *p* < 0.001.

## Data Availability

The data file of this study is publicly available on the Open Science Framework (OSF) at: https://osf.io/aw8e2/.
